# Crossmatch assays in transplantation: Physical or virtual?: A review

**DOI:** 10.1097/MD.0000000000036527

**Published:** 2023-12-15

**Authors:** Yermis Rocha, Andrés Jaramillo, Jorge Neumann, Katrin Hacke, Eduard Palou, Juan Torres

**Affiliations:** a University of Antioquia, Medellín, Colombia; b Department of Laboratory Medicine and Pathology, Mayo Clinic, Phoenix, AZ; c Transplant Immunology Laboratory, Santa Casa Hospital, Porto Alegre, Brazil; d Department of Immunology, Centre de Diagnòstic Biomèdic, Hospital Clínic de Barcelona, Barcelona, Spain.

**Keywords:** complement-dependent cytotoxicity crossmatch, flow cytometry crossmatch, single antigen bead, transplantation, virtual crossmatch

## Abstract

The value of the crossmatch test in assessing pretransplant immunological risk is vital for clinical decisions, ranging from the indication of the transplant to the guidance of induction protocols and treatment with immunosuppressants. The crossmatch tests in transplantation can be physical or virtual, each with its advantages and limitations. Currently, the virtual crossmatch stands out for its sensitivity and specificity compared to the physical tests. Additionally, the virtual crossmatch can be performed in less time, allowing for a reduction in cold ischemia time. It shows a good correlation with the results of physical tests and does not negatively impact graft survival.

Proper communication between clinicians and the transplant immunology laboratory will lead to a deeper understanding of each patient’s immunological profile, better donor–recipient selection, and improved graft survival.

## 1. Introduction

More than 50 years ago, Patel and Terasaki demonstrated for the first time that patients transplanted with a positive complement-dependent cytotoxicity crossmatch (CDCXM) had higher rates of hyper-acute rejection versus patients transplanted with a negative test.^[1]^ Since then, the crossmatch has become the gold standard for the detection of pretransplant antibodies against human leukocyte antigen (HLA). However, the low sensitivity of the CDCXM demanded the development of more sensitive techniques such as the flow cytometry crossmatch (FCXM) which allows the detection of complement-fixing or non-complement-fixing antibodies, lowers levels of donor-specific anti-HLA antibodies (DSA), and shows a better correlation with graft survival.^[1,2]^

Nowadays, the detection of anti-HLA antibodies by Single Antigen Bead (SAB) assays for immunological monitoring has allowed the use of the virtual crossmatch (VXM) test as a guide in the transplantation decision, showing similar results as the FCXM, allowing the reduction of cold ischemia times and delayed graft function.

Therefore, it is important to understand the characteristics, advantages, and limitations of each technique which will lead to a proper interpretation of a possible DSA (Table 1). In this review we will describe crossmatch tests in the context of kidney transplantation and how the results may impact transplant outcomes and the risk of antibody-mediated rejection (AMR).

**Table 1:** Comparison of crossmatching tests.

Crossmatch	CDCXM	FCXM	VXM
Target	Donor’s cells	Donor’s cells	Interpretation of results from SAB and donor HLA molecular typing
Type of antigens	Native HLA on cell membrane	Native HLA on cell membrane	Recombinant HLA proteins
Type of antibodies detected	Cytotoxic IgG/ IgM antibodies	IgG antibodies bonded on the lymphocyte membrane	IgG antibodies bound to HLA on the solid phase (Luminex)
Specific for anti- HLA antibodies	No	No	Yes
Interpretation	Subjective: Operator-dependent technique	Objective: Based on the difference between test serum MFI and negative control	Objective: Based on the MFI of specific antigens
Sensitivity	Low	High	High
Approximate processing time	5 hours	3 hours	≤1 hour
Predicting the clinical outcome of transplants	Hyperacute rejection	Acute rejection	Acute/chronic rejection

CDCXM = complement-dependent cytotoxicity, HLA = human leukocyte antigen, MFI = mean fluorescence intensity, SAB = single antigen bead, VXM = virtual crossmatch.

### 1.1. Complement-dependent cytotoxicity crossmatch: a starting point

The complement system is a crucial element of both innate and adaptive immunity, comprising over 30 proteins primarily synthesized in the liver. Complement activation leads to the formation of the membrane attack complex, resulting in the formation of pores in the cell membrane itself or pathogens, causing cell death and the release of inflammatory mediators that aid the immune response.^[3,4]^

Based on the principle of the crossmatch, in which the recipient’s serum’s reaction to a donor cell is tested, Terasaki et al described the CDCXM method. In this method, complement was added, and cell death was observed under the microscope.^[1]^ Thus, the CDCXM test involves using lymphocytes from donor peripheral blood, spleen, or lymph node samples, which are then incubated with the recipient’s serum, and rabbit serum complement is added to the mixture, and if there are donor-specific IgM or IgG antibodies present, the classical complement pathway is activated. The cell death can be observed under a microscope using a vital dye, and the American Society for Histocompatibility & Immunogenetics standardized ranking system is utilized based on the percentage of dead cells. The American Society for Histocompatibility & Immunogenetics scale score ranges from 1 to 8, with the cutoff point between negative and positive results being a 2 score (11–20% cell death).^[5]^

In the landmark study by Patel and Terasaki it was demonstrated that 80% of patients who underwent transplantation with a positive CDCXM experienced immediate kidney allograft failure, compared to only 15% of patients transplanted with a negative CDCXM.^[1]^ This made the CDCXM an essential and mandatory test for kidney pretransplant evaluation to avoid potential hyperacute rejection mediated by antibodies.^[1,6]^

Several technical modifications have been implemented to enhance the sensitivity and specificity of the CDCXM test. These include extended incubation times, lymphocyte isolation, washing steps, different incubation temperatures, the use of anti-human globulin (AHG), and treatment of sera with reducing agents like dithiothreitol. The latter is used to break the disulfide bridges of the IgM pentamers, generating monomers that are incapable of activating complement. This step is taken because IgM is not considered to have clinical significance in transplantation.^[7–10]^

Despite these modifications, the CDCXM still has certain limitations, such as:

- The CDCXM’s reliability depends on the viability of the donor cells. Nonspecific cell death caused by complement sensitivity can lead to false-positive results.
- Complement activation requires elevated antibody concentrations, which might affect the accuracy of the test.
- Additional processing steps are necessary to isolate B cells and differentiate between anti-HLA class I and class II antibodies.
- The CDCXM test exhibits low sensitivity in detecting anti-HLA class II antibodies, and the use of AHG does not improve this sensitivity due to nonspecific binding to fragment crystallizable receptors (FcR) on B cells.

Those factors can interfere with the assay’s efficacy and contribute to high interlaboratory variability.^[6,7,9,11]^ The histocompatibility laboratory and transplant clinicians need to be aware of these limitations and consider them when interpreting the results of the CDCXM.

The decrease in the use of CDCXM over the last decade, as reported in a 2022 study, might be attributed to the advancements and increased reliability of other crossmatching methods, particularly the FCXM, which has remained stable in its utilization.^[12]^ Despite the limitations and interlaboratory variability associated with CDCXM, its continued use may be driven by factors such as transplant clinicians’ preferences, their experience with the method, and their risk tolerance.^[12]^

### 1.2. Flow cytometry crossmatch: enhancing sensitivity

Patel and Terasaki initial study indicated that approximately 15% of kidney transplants with negative CDCXM results, experienced early graft loss and that despite the improvements made to the assay, it was necessary to develop a more sensitive technique that allowed the detection of lower DSA levels. For this reason, in 1983, Garovoy introduced the FCXM into clinical practice.^[2]^ Like the CDCXM, it consists of incubating donor cells and recipient serum, but no complement is added; instead, a fluorescently labeled anti-IgG antibody is used to detect the presence of donor-specific IgG antibodies that have bound to the surface antigens of lymphocytes, such as HLA. Additionally, the use of anti-CD3 and anti-CD19 or CD20 monoclonal antibodies allows for the discrimination of T and B lymphocytes subpopulations, respectively, which is beneficial for identifying antibodies against HLA class I (expressed by T and B lymphocytes) or HLA class II (expressed exclusively by B lymphocytes). One of the significant advantages of the FCXM is that it detects a broader range of immunoglobulins of clinical importance, including both complement-fixing and non-complement-fixing IgG antibodies^[7,10,13]^ Therefore, the FCXM test offers a higher sensitivity compared to the CDCXM, allowing the detection of lower DSA levels.

The FCXM test is analyzed using a flow cytometer, which allows us to obtain the results of the mean fluorescence intensity (MFI) emitted by the fluorescent dye-labeled anti-human IgG bound to donor cells incubated with recipient serum, compared to the MFI of the same cells treated with a negative control serum (usually a pool of healthy donor sera). The MFI difference between the recipient serum and the negative control, is usually referred as the median channel fluorescence (MCF) shift, and usually expressed as a ratio or delta of the MCF, which allows us to more objectively define the results when compared to the CDCXM visual interpretation and discriminating HLA class I and II antibodies in the same assay.^[7]^

Some laboratories have made modifications to FCXM protocols for the detection of lytic antibodies, like the CDCXM, for risk assessment in kidney transplant recipients, especially in patients with positive FCXM results.^[14–16]^ Nevertheless, this protocol is time-consuming, requires greater standardization, and a larger number of studies to demonstrate its superiority over current techniques.

Other modifications more popular to FCXM protocols, like the Halifax and Halifaster protocols, aim to reduce time and simplify their setup without compromising its quality or sensitivity.^[17]^ The Halifax protocol modifications resulted in a decrease of approximately 60% in processing time compared to the standard protocol. The principal changes introduced were the use of a 96-well U-bottom tray, a change in the cell-to-serum ratio, reduced incubation and wash times, and a higher incubation temperature. The Halifaster protocol improves lymphocyte purity to over 90%, reducing the number of target cells per reaction and increasing the sensitivity of the assay.^[17]^ Many transplant centers worldwide have adopted the Halifax protocol for routine clinical practice, with good interlaboratory concordance of the results.

Nowadays, current evidence strongly supports the greater sensitivity of the FCXM compared to CDCXM. Patients who undergo transplantation with a negative FCXM have been shown to experience higher graft survival rates and lower rejection rates compared to those transplanted with a positive FCXM and negative CDCXM.^[18]^

In a study carried out by Salvalaggio et al, they contrasted the results of AHG-CDCXM and FCXM in 230,995 transplant patients between 1999 to 2005 from the registry of the Organ Procurement & Transplantation Network.^[19]^ They found that patients transplanted with T−/B− FCXM were associated with an approximate 15% reduction in the relative risk of acute rejection compared to T−/B− AHG-CDCXM. Likewise, T−/B− FXCM was associated with a modestly improved 5-year graft survival among older recipients and recipients from deceased donors.^[19]^

Similar findings were evidenced by Graff et al with 66,594 patients with kidney transplants followed up to 5 years posttransplant.^[20]^ They found that recipients of living donors and T + FCXM, the graft survival was 6.2% and 12.4% lower versus transplanted with a T−/B− FCXM, at 1- and 5-years posttransplant, respectively. Patients transplanted with T−/B + FCXM presented a reduction in graft survival of 1.8% at 1-year posttransplant and 6.5% at 5 years posttransplant. The findings of the study are significant as they provide evidence that a positive FCXM result is associated with adverse effects on graft survival, and this risk continues beyond the peri-transplant period. The long-term risks associated with positive FCXM may be attributed to damage caused by memory effector cells.^[20]^

Also, Graff et al showed the impact on graft survival in transplants performed with negative CDCXM and positive FCXM results.^[21]^ The study was conducted on approximately 15,000 kidney transplant patients from living or deceased donors. They observed that patients transplanted from deceased donors with T + FCXM and T−/B− CDCXM had a 51% and 37% higher adjusted relative risk of graft loss at 1- and 5-years posttransplant, respectively. T−/B+ FCXM and T−/B− CDCXM transplants were not associated with an increased risk of early graft loss but were associated with a 42% higher adjusted relative risk of graft loss at 1 to 5 years after transplant. This study concludes the important prognostic implications of AMR of positive FXCM despite negative CDCXM.^[21]^

In general, the studies performed for comparing FCXM versus CDCXM indicate that approximately 15% of patients for primary grafts and up to 34% for regrafts will have a negative CDCXM with a positive FCXM.^[22]^ Likewise, early graft loss (<3 months) occurs approximately in 20% and 60% of primary grafts and regrafts, respectively, when FCXM is positive compared with 5% and 15%, respectively, when FCXM is negative.^[22]^

The clinical significance of FCXM has been well-established, even in cases where the CDCXM result is negative. However, it is crucial to acknowledge that the FCXM, like any diagnostic test, is not infallible and can yield false-positive or false-negative results. Lymphocytes express many surface antigens besides HLA molecules, and some of these antigens can bind antibodies circulating in the recipient’s serum, irrespective of their specificity.^[11]^

In FCXM have been described different causes of false-negative results, including: (a) low levels of HLA expression on donor cells, (b) excess number of cells, (c) low serum volume (which is generally a bad cell-to-antibody ratio), (d) low DSA levels, (e) low purity of the lymphocytes, and (f) high background in the negative control serum. False-positive results may be due to: (a) IgG binding to FcR in B cells, (b) low background in the negative control, (c) insufficient washing after incubations with antibodies, (d) autoantibodies, and (e) the use of therapeutic antibodies such as anti-thymocyte globulin, rituximab (anti-CD20), alemtuzumab (anti-CD52), basiliximab (anti-CD25), and daclizumab (anti-CD25).^[10,11]^

Correlating crossmatch results with patient history, sensitizing events, and DSA history is important when interpreting possible false-positive or false-negative scenarios. Therefore, an adequate analysis will allow us to define if they are DSAs and, therefore, a more precise immunological risk based on the FCXM.

### 1.3. Virtual crossmatch: better than the physical crossmatch?

The VXM is not a laboratory test per se; rather, it relies on the interpretation of previous results from the recipient’s anti-HLA analysis using Single Antigen Bead (SAB) assays and donor HLA molecular typing. As a result, can determine the presence of DSA and the immunological risk. The SAB assay uses the Luminex system and is based on the use of fluorescently labeled polystyrene microspheres. These microspheres have recombinant HLA molecules attached to their surface, and each bead carries a single HLA antigen molecule. In this assay, the recipient serum is incubated with the beads; then, a secondary fluorescence labeled anti-IgG is added, and subsequently analyzed by Luminex cytometer; the results of anti-HLA antibody binding to the beads are expressed as MFI.^[23]^

Although the MFI is a numerical value, the SAB is not a quantitative test. The MFI represents the relative immunofluorescence of the antigen-antibody binding, without a reference standard. However, MFI values have been used in clinical practice and at the laboratory level as a semiquantitative test, useful for monitoring patients and predicting the clinical outcome of the transplant.^[24–26]^

Determining the presence of anti-HLA antibodies by SAB in patients on the transplant waiting list helps us define not only the risk of AMR but also establish a criterion for prioritization when assigning a potential donor. This is achieved through the calculated panel-reactive antibody, which represents the calculated probability of the recipient’s antibody profile being positive against the HLA types of a pool of randomly chosen donors.

The VXM results have shown the ability to predict the results of the physical crossmatch test with a high level of concordance. Additionally, research has demonstrated that patients transplanted with a negative FCXM result, but a positive VXM results are at an elevated risk of AMR and experience a shorter graft survival.^[27–35]^

On the other hand, it has also been observed that in patients with DSA detectable by the SAB in the presence of a negative FCXM, the immunological risk is minimal in the intermediate term, and there is no significant impact on acute rejection rates.^[36,37]^ These patients did not require desensitizing therapy, suggesting that these antibodies should not represent a barrier to transplantation. Even though patients may have a higher rate of acute rejection in the first year after transplantation, this does not translate into lower graft survival in the intermediate term.^[36,37]^

In a study published in 2010, Taylor et al describe their 10-year experience selectively omitting physical crossmatches in deceased-donor kidney transplant patients.^[38]^ They found no differences between those who underwent physical testing versus those who did not in terms of delayed graft function (34% vs 33%, respectively), acute rejection in the first 12 months posttransplant (27% vs 25%, respectively), and graft/patient survival.^[38]^

In 2020, Roll et al published the experience of patients who underwent kidney transplantation between 2014 and 2017 at a hospital in San Francisco, CA.^[39]^ During this period, a total of 254 kidneys were transplanted based on the results of VXM. Among these cases, 84.6% (215) were transplanted without a physical crossmatch, and within this group, 54.9% of the patients had a calculated panel-reactive antibody level of ≥99%. Among those who were transplanted solely based on VXM results, there were no instances of hyperacute rejection. Only one case of acute rejection occurred in the first year after transplantation, indicating a potential immunological memory. Notably, there were no significant differences in graft function compared to those who underwent transplantation with physical crossmatching tests.^[39]^

Indeed, the centers that rely solely on the results of VXM for transplantation have recognized the importance of strict immunological follow-up for their patients. This follow-up involves conducting SAB testing regularly, typically every 3 months, or more frequently if patients have received blood transfusions or experienced systemic infections. According to the standards of the European Federation for Immunogenetics, version 8.0, the patients eligible for VXM transplantation are those who^[40]^:

- There have been no potential sensitizing events since the last serum sample screened.
- At least 2 different samples must have been tested and at least one serum testing result obtained within the previous 3 months must be included.
- Acceptable and/or unacceptable mismatches have been clearly defined and documented.
- If a prospective crossmatch is omitted from an alloimmunized recipient, the method of antibody identification must rely on SAB technology.

Considering the SAB assay currently would be the technique with the highest sensitivity for the detection of anti-HLA antibodies. If we were to use this technique as the gold standard and for orientation purposes (as mentioned, it is a semi-quantitative test), we could detect anti-HLA antibodies with an MFI ≤ 1000 (depending on the specific commercial supplier of the Luminex assay). The FCXM would be positive when the SAB start from an MFI = 2500 (also depending on the locus, with a higher MFI for HLA-C, DQB1, or DPB1), and the CDCXM would be positive starting from an MFI = 8000–10,000. This information is useful for correlating the results obtained from the crossmatch tests and interpreting them in a clinical context.

**Figure 1.**
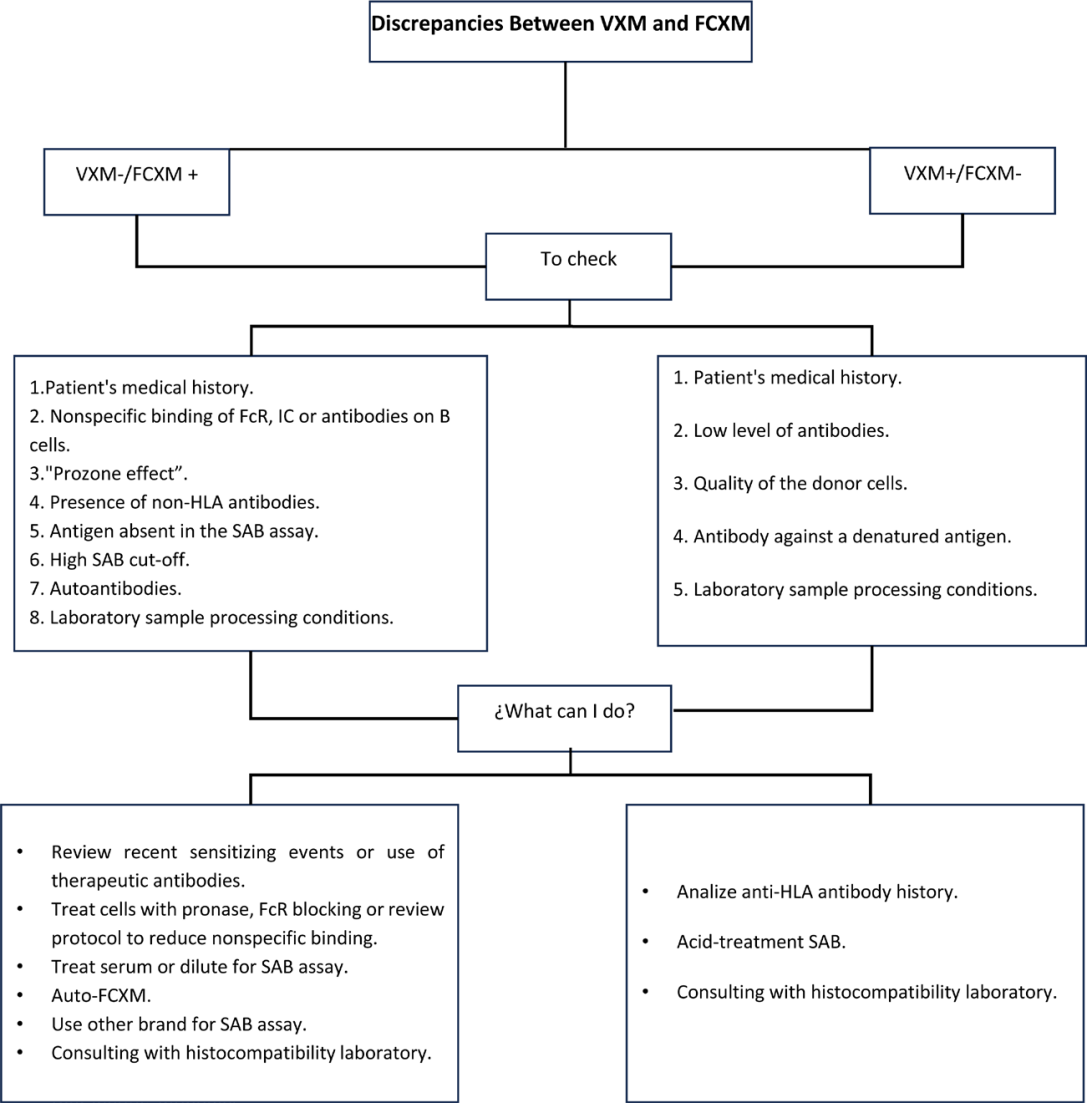
Discrepancies between VXM and FCXM.

Approximately 20% of cases may have a positive FCXM and a negative VXM, with 90% of these cases being positive solely for B cells without the presence of DSA in the patients.^[29]^ While most of these cases may be false-positive FCXM results, the possibility of an antibody being present cannot be ruled out. Understanding the limitations of the SAB, using a processing protocol to eliminate interferences, and performing additional crossmatching tests can be of great value in resolving these discrepancies. Among the possible causes of these discrepancies are (Fig.1):

- The binding of immune complexes or antibodies to the FcR or complement receptors on B cells: While it has been described that treatment with pronase on B cells can decrease this phenomenon, its use must be careful and properly standardized. Pronase cleaves various proteins on the cell surface nonspecifically, which can lead to false positives or negatives due to the exposure of cryptic antigens or degradation of HLA, respectively.^[41,42]^
- High levels of antibodies can lead to complement activation with C1 deposition on the beads, presence of IgM, immune complexes, intravenous immunoglobulin, thymoglobulin, and other factors in the patient’s serum, which may interfere with the binding of the secondary antibody or the binding of IgG to the bead, resulting in false negative results. This phenomenon is the so-called prozone effect.^[24,26,43]^ Pretreatment of sera with EDTA, heat, dithiothreitol, or dilutions has been shown to decrease this effect.^[23,44,45]^
- Presence of non-HLA antibodies: Although this issue is controversial, previous studies have shown successful transplant results under this discrepancy, so non-HLA antibodies are not considered a contraindication for transplantation.^[29]^ In the recent review of the STAR 2022 (Sensitization in Transplantation: Assessment of Risk), there is a strong emphasis on the need for improved and expanded validation of the tests used for measuring non-HLA antibodies. Adequate monitoring of the appearance of these antibodies and investigating synergistic effects between DSA and non-HLA antibodies are also highlighted. The goal is to achieve a better understanding of their contribution to the transplant outcome.^[46]^
- Although uncommon, it is possible that the patient may be reacting against a rare antigen that is not present in the SAB assay.^[29]^
- High SAB cutoff: While the cutoff varies from center to center, most institutions that omit physical crossmatching consider an MFI between 1000 to 2000 as low immunological risk. They may also establish different cutoffs for specific loci like HLA-C, DQ, and DPB1 since these are overexpressed in the SAB assay. Additionally, for patients with previous transplants, antigens from their previous donor may be excluded regardless of the MFI level.^[27,35,39,47]^ It is important to note that the technical cutoff is established in the histocompatibility laboratory based on validation processes and internal analyses conducted in each run. This cutoff is not fixed, and each transplant center must assess the risk for each patient based on the immunosuppressive regimen, the patient’s immunological history, clinical condition, and previous experiences. Hence, ongoing communication between clinicians and laboratory personnel is vital. While there are general parameters to guide decision-making, every patient should be treated individually to provide the best option for a donor-recipient match.
- Although it has been suggested that a SAB assay could be falsely negative due to “dilution” of the antibody, caused by a shared epitope among multiple beads, especially when targeting a public epitope like Bw4 and Bw6,^[23,48]^ this hypothesis was recently debated by Claisse et al.^[49]^ They assessed the impact of MFI dilution using beads isolated from only one HLA allele containing the target eplet. Their results indicate no evidence of clinically significant MFI dilution for the studied eplets. Therefore, this hypothesis should not currently be regarded as a limitation of SAB assays for evaluating the strength of patient antibodies and, consequently, as an additional immunological risk factor.^[49]^ While Claisse’s study extensively examines this phenomenon, it would be interesting to evaluate other eplets, replicate the assay with a different commercial kit, and employ live cells to validate this phenomenon.

Another discrepancy that can be found in approximately 5% of cases is having a negative FCXM and a positive VXM, the possible causes may be:

- A weak antibody that does not reach a positive FCXM: In general, studies have shown that a positive FCXM can be obtained with MFI ≥ 2500.^[31,50]^ However, it is worth noting that the success of any physical crossmatching test relies on the quality of the donor cells, and it is known that HLA expression can vary depending on whether the donor is living or deceased, as well as the source of the cells, whether from peripheral blood, spleen, or lymph node. Certain medications, such as statins and steroids, can decrease HLA expression.^[50–54]^ Usually, low HLA expression can result in false negative results.
- An antibody against a denatured antigen can give a false positive in SAB assays. These antibodies bind to cryptic epitopes that are not normally accessible to antibodies under normal conditions. Therefore, it has been proposed that they are not harmful and do not contraindicate transplantation.^[43,55–58]^

One important point to highlight, which helps us better understand the results of the SAB assay and the previously mentioned discrepancies, is to perform an epitope analysis as an approach to estimate the sensitizing eplet based on the serum positivity pattern. The HLAMatchmaker as well as other algorithms such as the Predicted Indirectly Recognized HLA Epitopes (PIRCHE) score, have been described as methods to assess molecular differences in HLA and predict an immune response.^[59–61]^ Preliminary work has already been validated in larger cohorts and the initial results have supported evidence that epitope matching is associated with de novo DSA formation and decreased graft survival. However, our understanding of epitope matching and its relationship to clinical outcomes is limited. We still need to determine the most effective way to apply the findings from the population level to the individual patient’s care. Therefore, there is a need to seek consensus on the definition and interpretation of epitope matching. It is important to note that reactivity against a specific eplet does not necessarily mean that all alleles carrying that eplet are considered unacceptable. It is known that the amino acids surrounding the eplet play a role in the immunogenicity. Assessing the relative immunogenicity of individual epitopes is essential to prevent the rejection of suitable organs due to epitope mismatches that have no clinical relevance.^[60,62,63]^

When dealing with a recipient with anti-HLA antibodies detected by SAB, it is useful to correlate these antibodies with sensitizing events that can explain the pattern of positivity observed. In many cases, the information about the sensitizing event may be incomplete, leading to potential false positives. In such situations, the physical crossmatching test can be valuable in determining whether these antibodies are truly donor-specific.

Finally, it is usually the case that histocompatibility laboratories do not receive payment for performing VXM. However, on November 2, 2022, the final 2022 Medicare Physician Fee Schedule was released in the US, which included pathology consultation services codes and values developed by the College of American Pathologists and the American Medical Association. These codes are expected to enhance the physician service reporting and billing process for pathologists due to improved instructions and a basis for code selection, either “Time” or “Medical Decision Making”. Several transplant centers in the US are now using these pathology clinical consultation codes to receive reimbursement for VXM consultations.^[64]^ The initial VXM may be performed by a Ph.D. laboratory director but the second and final report must be signed by a board-certified pathologist.

The new code family is based on the degree of complexity and the time of service, broken down into 20-minute increments for each code, with an additional code that includes an extra 15 to 30 minutes. The paper by Karcher showcases the new codes for Pathology clinical consultation established in US, such as^[64,65]^:

- Code 80503: For a clinical problem, with limited review of patient’s history and medical records and straightforward medical decision making (MDM).
  When using time for code selection, 5 to 20 minutes of total time is spent on the date of the consultation.
- Code 80504: For a moderately complex clinical problem, with review of patient’s history and medical records and moderate level of MDM.
  When using time for code selection, 21 to 40 minutes of total time is spent on the date of the consultation.
- Code 80505: For a highly complex clinical problem, with comprehensive review of patient’s history and medical records and high level of MDM.
  When using time for code selection, 41 to 60 minutes of total time is spent on the date of the consultation.
- Code 80506: Prolonged service, each additional 30 minutes (list separately in addition to code for primary procedure).

A pathology clinical consultation used for the VXM is a service rendered by a pathologist in response to a request from a physician or other qualified healthcare professional, evaluating histocompatibility laboratory findings and other relevant clinical or diagnostic information that gives a risk assessment of a transplant between a particular donor-recipient pair. Reporting histocompatibility laboratory findings or other relevant clinical or diagnostic information without a medical interpretive judgment is not considered a pathology clinical consultation.^[64]^

## 2. Conclusions

Understanding the limitations of each test is of vital importance to adequately correlate laboratory findings. Equally important is the analysis of the patient’s historical results in SAB assay to gain a deeper understanding of the patient’s immunological complexities and assess the risks associated with a potential donor. Atypical or discrepant cases present an excellent opportunity for discussions between the histocompatibility laboratory and transplant clinicians. These discussions enrich the understanding of immunological complexities and allow for fine-tuning each algorithm based on the patient’s needs.

A continuous and open communication between the histocompatibility laboratory and medical staff improves overall patient care by ensuring a thorough evaluation of immunological risks and making informed decisions in the donor-recipient matching process. This leads to better transplant outcomes and long-term success.

## Author contributions

**Conceptualization:** Yermis Rocha.

**Formal analysis:** Yermis Rocha, Andrés Jaramillo, Jorge Neumann, Katrin Hacke, Eduard Palou, Juan Torres.

**Project administration:** Yermis Rocha.

**Supervision:** Yermis Rocha.

**Validation:** Yermis Rocha, Andrés Jaramillo, Jorge Neumann, Katrin Hacke, Eduard Palou, Juan Torres.

**Writing – original draft:** Yermis Rocha.

**Writing – review & editing:** Yermis Rocha, Andrés Jaramillo, Jorge Neumann, Katrin Hacke, Eduard Palou, Juan Torres.
